# Knowledge and Attitudes Concerning Antibiotic Use and Resistance among the Public in Pulau Pinang, Malaysia

**DOI:** 10.21315/mjms2018.25.6.15

**Published:** 2018-12-28

**Authors:** Nur Ashila Azleen Ab Halim, Chee-Tao Chang, Huan Keat Chan, Mohamed Azmi Hassali, Ahmed Nouri

**Affiliations:** 1 School of Pharmaceutical Sciences, Universiti Sains Malaysia, 11800 USM, Pulau Pinang, Malaysia; 2 Clinical Research Centre, Raja Permaisuri Bainun Hospital, 30450 Ipoh, Perak, Malaysia; 3 Clinical Research Centre, Sultanah Bahiyah Hospital, KM 6, Jalan Langgar, 05460 Alor Star, Kedah, Malaysia

**Keywords:** anti-bacterial agent, knowledge, attitude, Malaysia

## Abstract

The aim of this cross-sectional study was to evaluate the knowledge and attitudes concerning antibiotic use and resistance among members of the local community in Pulau Pinang, Malaysia. The study was conducted among 326 residents of the Jelutong district, Pulau Pinang state, from August to October 2013. A self-administered five-part questionnaire was used for the data collection. The respondents exhibited inadequate knowledge of antibiotics in general. Approximately 80% of them did not know the indications for antibiotic use, while 76% believed that antibiotics were useful in terms of resolving viral fever. Additionally, 52.6% believed that antibiotics could be used to treat all types of infections. Inadequate knowledge of antibiotic resistance was also evident among the respondents, since 72.9% of them did not agree that resistant bacteria can spread from human or animal to human, while 32% were unaware that bacteria can develop resistance to antibiotics. More than 60% of respondents admitted that they took antibiotics in order to accelerate their recovery from illness, while 34.8% claimed that they only stopped taking antibiotics when they felt better. The findings hence indicate that most respondents had poor knowledge and attitudes concerning antibiotic use and resistance, which suggests the need for more community-based educational campaigns designed to improve the public’s knowledge and attitudes regarding antibiotics.

## Introduction

To date, a wide range of viral and commonly self-resolving diseases, including the common cold, sinusitis, sore throat and bronchitis, have been treated with antibiotics worldwide despite the lack of efficacy of such a treatment modality. The use of antibiotics has also often been shown to not be supported by culture and sensitivity testing, nor is it always in line with the recommendations governing clinical practice (1, 2). According to the World Health Organization, 80% of antibiotic consumption takes place in the community, with 20%–50% of such medications being inappropriately used (3). However, the level of knowledge and attitudes concerning antibiotic use vary across different countries and communities. For example, a systematic review revealed that approximately half of all respondents reported discontinuing antimicrobial treatment when they felt better (4), although such a practice was reported by only 15.6% of respondents in Turkey (2). Further, self-prescription and the excessive use of antibiotics, such as penicillin and erythromycin, have been reported to be fairly prevalent in developing countries (5, 6).

In Malaysia, a poor level of knowledge regarding antibiotic use has previously been observed. Indeed, more than 80% of respondents in a study conducted in Putrajaya believed that antibiotics could cure viral infections (7, 8). The majority of them also expected to receive antibiotics to treat a cough or cold. Furthermore, consistent with the global trend, a considerable proportion of respondents reported that they would stop the antibiotic treatment if they felt better. This is an issue of significant concern, since poor knowledge and attitude have been found to be associated with the high usage of antibiotics in the community, as well as with a higher rate of antimicrobial resistance (9).

Antimicrobial resistance has been defined as the ability of bacteria and other microorganisms to resist the effects of an antibiotic to which they were once sensitive (10). In addition to the well-known methicillin-resistant *Staphylococcus aureus*, other types of microorganisms, including *Escherichia coli*, *Pseudomonas aeruginosa* and *Klebsiella pneumonia*, have been reported to exhibit resistance to commonly used antibiotics (11). As a consequence, less conventional antibiotics, which are generally more costly, are now widely used by health-care providers. Such a practice has unavoidably led to a significant increase in expenditure on drugs and hospitalisation worldwide. In the United States alone, extended hospitalisation due to antibiotic-resistant infections for a period ranging from 6.4 to 12.7 days has resulted in an additional cost of up to USD29,069 per patient, which has led to estimated additional health-care costs of USD20 billion and an annual loss in productivity of USD35 billion (6).

Although several prior studies have highlighted concern about the poor knowledge and attitudes exhibited by Malaysians with regards to antibiotic use, their findings were limited to patients who attended hospital (7, 8). Hence, this study was conducted beyond the hospital setting, with the aim being to assess the knowledge and attitudes concerning antibiotic use and resistance among the general public.

## Methodology

A cross-sectional survey using a validated self-administered questionnaire was conducted among the population of the Jelutong district, Pulau Pinang state, from August to October 2013. The questionnaire was adapted from several studies (9, 12, 13), and it comprised five parts: (i) demographic characteristics, (ii) history of antibiotic use over the past three months, (iii) knowledge of antibiotics (five statements with ‘yes’ and ‘no’ answer options), (iv) attitude regarding antibiotic use (six statements concerning storage, compliance, self-medication and expectation, with ‘agree’, ‘neutral’ and ‘disagree’ answer options), and (v) knowledge of antibiotic resistance (seven statements concerning the role of antibiotics, adverse effects and antibiotic resistance, with ‘yes’ and ‘no’ answer options).

The face and content validation of the questionnaire was performed by two academicians from the School of Pharmaceutical Sciences, Universiti Sains Malaysia. The initial version of the questionnaire was finalised following a meeting attended by all the investigators and invited academicians. The questionnaire was initially developed in English, and it was subsequently translated into the Malay language by a certified translator based on the recommendations of the United States Census Bureau (14). It was then back translated into English by a bilingual academician. Both versions were proven to be as semantically close as possible before being used in the data collection process.

The questionnaire was pilot tested with 30 residents of the Jelutong district. The reliability of the questionnaire was confirmed by its Cronbach’s α value of 0.647 (15). No further modifications were made after the pilot test, and the tool was subsequently used for data collection. The sample size was calculated using the Sample Size Calculator for Prevalence Studies (16). A minimum sample size of 264 was required, taking into account the expected prevalence of inappropriate knowledge in the pilot study (22%), the 95% confidence interval and the need for 5% precision. In order to account for a 20% non-response rate, a total of 333 participants were required for this study (8).

The respondents were recruited using the convenience sampling method. Only those who were over 18 years of age, who understood English or the Malay language, and who had previously heard of the term ‘antibiotic’ were included in this study. All the respondents were approached at their residences, community centres or commercial centres. Written consent was obtained from all respondents before the study procedures were initiated. Each respondent was given 10 to 15 min to complete the questionnaire.

The Statistical Package for the Social Sciences (SPSS) for Windows version 20.0 (IBM, New York) was used to analyse the data. The respondents’ demographic characteristics, along with their knowledge and attitudes concerning antibiotics use and resistance, were descriptively reported. The level of statistical significance was set at *P* < 0.05.

## Results

Of the 350 questionnaires distributed, 326 were completed (92.9% response rate). The majority of respondents were female (63.8%), above 40 years of age (72.1%), Malay (91.1%) and had received at least a secondary education (69.7%). One hundred and nine (33.4%) of them worked in the private sector, while 20% of respondents were civil servants and housewives, respectively. The majority of them had a monthly household income of below RM2,000 (62.6%) (Table 1). The vast majority of respondents (83.7%) were found to incorrectly believe that antibiotics could be used treat viral infections. Furthermore, 76% of respondents believed that antibiotics could alleviate a viral fever. It was also found that 52.6% of them believed that antibiotics could be used treat all types of infections (Table 2).

In terms of their attitudes towards antibiotic use, 207 (63.7%) respondents admitted that they had taken antibiotics in order to accelerate their recovery from illness. Further, nearly half of all respondents reported either keeping antibiotics for future use or expecting doctors to prescribe antibiotics when they were sick. Their inadequate knowledge of antibiotic resistance was also evident, since 40.6% and 45.8% of respondents disagreed that bacteria and viruses, respectively, could develop resistance to antibiotics (Table 2).

## Discussion

To the best of our knowledge, this is the first study to assess the knowledge and attitudes of the public with regards to antibiotic use in Malaysia. The findings could, therefore, prove helpful for health-care professionals and policymakers alike in terms of better understanding the level of knowledge of the local community and their antibiotic-taking behaviours, which could subsequently help with the development of strategies for tackling the issue of inappropriate antibiotic use.

Overall, the respondents demonstrated insufficient knowledge and poor attitudes concerning antibiotic use. The findings suggest that the majority of respondents in this study did not clearly understand the purpose of taking an antibiotic. The majority of them also believed that antibiotics could be used to treat all types of infections, including viral infections. Such findings are consistent with those of hospital-based studies conducted in Malaysia (7, 8), although the proportion of respondents who exhibited limited knowledge was higher than the proportions reported in other countries (2, 17). As previously reported, poor knowledge regarding the role of antibiotics has been found to be a predictor of both inappropriate antibiotic use and antibiotic resistance (9, 17, 18). Hence, there exists a clear need to improve the public’s awareness of the possible consequences of excessive antibiotic use and antibiotic resistance (9, 19).

It is also noteworthy that more than half of all respondents in this study expected antibiotics to be prescribed by doctors if they were sick, even though the proportion of respondents who reported such expectations was relatively low when compared with proportions seen in previous studies (7, 8). It has been established that unnecessary or excessive prescribing is more likely to occur due to overly high expectations and pressure from patients (2, 9, 12). Additionally, when compared with previous observations in other areas of Malaysia (7, 8), we found that the phenomenon of respondents discontinuing their treatment when they felt better was more common in Penang. It is imperative to note here that incomplete antibiotic treatment could increase microbial resistance to antibiotics (9, 19). Thus, in order to enhance the public’s awareness of the consequences of antibiotic resistance, educational strategies that address the public’s knowledge gap, including educational campaigns and the use of promotional materials, are warranted (9, 19)

In is important to note that there were several limitations to this study. First, the convenience sampling method might have unavoidably caused selection bias during the recruitment of respondents. Furthermore, the generalisability of the findings is likely to be limited, since the study was conducted in only one state in Malaysia. Thus, a nationwide survey adopting a random sampling method is required in the future to provide further insight into the public’s awareness of antibiotic-related issues in Malaysia.

## Conclusion

Most respondents in this study were found to have inadequate knowledge and attitudes with respect to both antibiotic use and antibiotic resistance, which suggests that more educational campaigns are warranted in order to improve the public’s awareness and promote the rational use of antibiotics in Malaysia.

## Figures and Tables

**Table 1:** Demographic characteristics of the respondents

Demographic characteristics		Results	

		Frequency (*n*)	Percentage (%)
Age (years)	18–30	27	8.3
	31–40	64	19.6
	41–50	74	22.7
	51–60	85	26.1
	60 or above	76	23.3
Gender	Male	118	36.2
	Female	208	63.8
Race	Malay	297	91.1
	Chinese	22	6.7
	Indian	7	2.2
Educational level	Primary	76	23.4
	Secondary	186	56.9
	College/university	64	19.7
Employment status	Private	109	33.4
	Government	67	20.6
	Housewife	68	20.9
	Retired	28	8.6
	Unemployed	54	16.5
Monthly household income	< RM500	48	14.7
	RM500–1000	55	16.9
	RM1000–2000	101	31.0
	RM2000–4000	80	24.5
	> RM4000	42	12.9
Healthcare-related occupation	Yes	62	19.0
	No	264	81.0
With family members working in the healthcare sector	Yes	93	28.5
	No	233	71.5

**Table 2:** Knowledge of and attitude in antibiotic use and resistance

No	Questions	Areas	Correct Response (*n*)	Correct Response (%)
Knowledge of antibiotics				
1.	Antibiotics are able to treat viral infections.	Role of antibiotics	53	16.3
2.	Antibiotics can alleviate viral fever.		78	24.0
3.	Antibiotics can treat all types of infections.		154	47.4
4.	Antibiotics may cause allergic reactions.	Adverse effects	261	80.3
5.	Antibiotics do not cause side effects.		94	28.9
6.	Bacteria can develop resistance to antibiotics.	Antibiotics resistance	193	59.4
7.	Overuse of antibiotics can cause bacteria to develop resistance against antibiotics.		197	60.6
8.	Viruses can develop resistance to antibiotics.		149	45.8
9.	The use of antibiotics can increase the resistant of viruses.		135	41.5
10.	Antibiotic-resistant bacteria can spread (from human/animals to human).		88	27.1
Attitude towards antibiotic use				
1.	I throw away the unused antibiotics.	Storage	152	46.8
2.	I take the antibiotics according to the instructions provided by doctors/ pharmacists/on the label.	Compliance	313	96.4
3.	I stop taking antibiotics when my symptoms disappear.	Compliance	113	34.8
4.	I usually give my antibiotics to my family members when they are sick.	Self-medication	276	84.9
5.	When I do not feel well, I take antibiotics to speed up recovery.	Self-medication	207	63.7
6.	I expect antibiotics to be prescribed by my doctor if I am sick.	Expectation	162	49.8
